# Pettiness: Conceptualization, measurement and cross-cultural differences

**DOI:** 10.1371/journal.pone.0191252

**Published:** 2018-01-31

**Authors:** Reuben Ng, Becca Levy

**Affiliations:** 1 Lee Kuan Yew School of Public Policy, National University of Singapore, Singapore, Singapore; 2 Geriatric Education and Research Institute, Singapore, Singapore; 3 Social and Behavioral Sciences, School of Public Health, Yale University, New Haven, Connecticut, United States of America; Pennsylvania State University College of Medicine, UNITED STATES

## Abstract

Although pettiness, defined as the tendency to get agitated over trivial matters, is a facet of neuroticism which has negative health implications, no measure exists. The goal of the current study was to develop, and validate a short pettiness scale. In Study 1 (*N* = 2136), Exploratory Factor Analysis distilled a one-factor model with five items. Convergent validity was established using the Big Five Inventory, DASS, Satisfaction with Life Scale, and Conner-Davidson Resilience Scale. As predicted, pettiness was positively associated with neuroticism, depression, anxiety and stress but negatively related to extraversion, agreeableness, conscientiousness, openness, life satisfaction and resilience. Also, as predicted, pettiness was not significantly related to physical functioning, or blind and constructive patriotism, indicating discriminant validity. Confirmatory Factor Analysis in Study 2 (*N* = 734) revealed a stable one-factor model of pettiness. In Study 3 (*N* = 532), the scale, which showed a similar factor structure in the USA and Singapore, also reflected predicted cross-cultural patterns: Pettiness was found to be significantly lower in the United States, a culture categorized as “looser” than in Singapore, a culture classified as “tighter” in terms of Gelfand and colleagues’ framework of national tendencies to oppose social deviance. Results suggest that this brief 5-item tool is a reliable and valid measure of pettiness, and its use in health research is encouraged.

## Introduction

Many individuals have experienced pettiness or the negative appraisal of a trivial event, such as getting annoyed while driving behind a slow car. Pettiness or “sweating the small stuff” [1] has gained attention in pop psychology, and self-health books [2], perhaps because of its potential pervasive reach in daily life. In contrast, it has been largely ignored in research. This may be due to the absence of a pettiness measure. The goal of the following set of studies was to develop a brief and valid measure.

There is reason to believe that pettiness may be associated with worse health. Pettiness is thought to be a component of neuroticism, the tendency to feel negative about both major and minor events, which has been identified as a public health concern [3]. Many studies found that it is associated with negative physical outcomes including poorer physical function [4], higher incidence of disability, chronic illness, and premature mortality [5]. Neuroticism has also been associated with negative psychological consequences [6]. In 2007, individuals in the top quartile of neuroticism scores incurred an excess healthcare cost of more than $1.3 billion in Holland [7].

Despite the considerable research on the negative health associations with neuroticism, the facets that make it up have been little studied. As Lahey [3] points out, neuroticism is a “heterogeneous trait consisting of multiple facets that are highly correlated but partially distinct” (p. 241). Disentangling the various aspects of neuroticism is paramount given its potentially insidious consequences. In the following three studies, we developed and validated a pettiness scale which could be used to disentangle the degree to which health is influenced by negative appraisal of minor events.

The first study developed the items in the pettiness scale and tested it against other constructs for convergent (e.g., depression, anxiety, stress) and divergent (e.g., physical functioning) validity. Thereafter, the second study tested the stability of the pettiness scale in a new sample. The third study validated the structure of the scale across two cultures with the aims of establishing cultural invariance, and examining predicted differences in pettiness.

The five-factor model of personality offers a helpful framework to test the construct validity of pettiness. It is a trait approach that provides a parsimonious taxonomy to define disparate personality constructs [8,9]. Besides neuroticism, the other four factors are extraversion, defined as an enthusiastic approach towards one’s social world; agreeableness, defined as the propensity to be accommodating in social situations; conscientiousness, defined as a propensity to be achievement-orientated; and openness, defined as an inclination towards new ideas and situations.

To establish construct validity, pettiness is hypothesized to positively correlate with neuroticism because it is predicted to be a component of neuroticism. Conversely, pettiness is hypothesized to negatively correlate with the other four personality factors because the incessant focus on trivial matters contrasts with the accommodating features of agreeableness, and the goal-achievement focus of conscientiousness. Pettiness is also diametrically different from the inclination towards new/uncertain situations of openness and the energy directed at one’s social world (extraversion).

Increasing the scope and rigor for construct validity, we further hypothesized that pettiness is positively associated with internalizing conditions such as depression, anxiety and stress and negatively related with life satisfaction and resilience. To test for discriminant validity, we hypothesize that pettiness is not related to physical functioning, defined as the ability to perform activities of daily living (ADL). While personality may influence ADL in sub-groups such as centenarians [10] and elders with depression [11], there is no evidence for significant associations between personality and one’s ability to perform ADL in normal populations. Further, discriminant validity will be tested through the non-significant relationship between pettiness, blind and constructive patriotism. Both constructs have no theoretical reason to be linked, and there is no empirical evidence to suggest the contrary.

With respect to cross cultural differences, we hypothesized that pettiness will be higher in a “tight culture,” characterized by strong social norms and low patience for deviant behavior, and lower in a loose culture, which is defined as a culture with weak social norms that tolerates deviations [12]. We drew on Gelfand and colleagues’ [12] survey of 33 nations to identify two countries for Study 3, one from the top quartile of tightness scores (Singapore), and another from the bottom quartile of tightness scores (USA). Given the features of a tight and loose culture, we expect Singaporeans to score higher in pettiness than Americans. Specifically, given the higher adherence to social norms, Singaporeans may be more provoked and agitated when others do not conform to trivial social norms.

In addition, studies on prevention-focused and promotion-focused cultures further support the hypothesis that residents of Singapore would be higher in pettiness than those in the United States. Prevention-focused cultures—that include Singapore [13]—are detail-orientated and place great emphasis on the absence of *negative* outcomes, perpetuating a state of vigilance where individuals are careful not to make mistakes [14]. Such a state of vigilance may promote Singaporeans’ focus on trivial matters and increase the risk of getting agitated over them. The United States, on the other hand, has been described as a promotion-focused society where the focus is on nurturance, and the presence of *positive* outcomes [15]. Besides documenting this potential cross-cultural difference, Study 3 examined whether pettiness shows an equivalent structure across two highly different cultures.

## Study 1a: Initial validation, exploratory factor analysis, and construct validity

The aim of Study 1a was to develop the scale items and examine the construct validity of the new scale.

### Methods

#### Scale construction

We derived an initial pool of 20 items from discussions with five experts working in the general area of psychosocial epidemiology, and personality psychology. Thereafter, the item pool was sent to another 10 experts conducting research on psychosocial factors of health who provided open-ended feedback on item wording, questionnaire flow, and items they would add and discard. These individuals were chosen based on their expertise in personality science, psychosocial epidemiology and psychopathology, and availability to provide feedback which are important to establish face validity of the items—the extent to which the questions measured the construct. Importantly, the expert panel members were able to recognize that the construct being measured was distinct from anger, hostility, and neuroticism, thus supporting face validity. Based on expert feedback, six items were retained. Experts were informed that their opinions may be included in an aggregated form.

No behavior-specific items were included because experts opined that the triviality of a situation is not universal. A behavior that may seem trivial to one individual or cultural group may not be trivial to another individual or cultural group. Moreover, triviality may differ from situation to situation even for a person who is high on pettiness. Endorsing a particular behavior such as “getting offended by someone who cuts in front of us in traffic” may measure not only pettiness but anger and anxiety.

#### Participants

A sample of 1720 students from three universities in Singapore participated in the study as part of a larger project on social attitudes. As the language of instruction and communication in Singapore is English, the study was conducted in English. Within each broad academic discipline (for every institution)—Natural Science, Social Science and Humanities—two introductory courses were randomly selected. Their professors sent e-mail invitations to all students enrolled in these classes. Introductory courses of 100–200 students were targeted because students came from many different academic disciplines, and reflected the diversity of students in each institution. Response rates were 54.3%, 57.1%, and 55.8% for the respective universities.

Participants were 56% female, 70% Chinese (16% Malay; 10% Indian; 4% others) and ranged from 20 to 25 years (*M* = 22.7; *SD* = 2.65). Surveys were administered either in a paper version during class time or electronically via a website. There were no significant differences in demographics (gender, *p* = .91; race, *p* = .89), and pettiness (*p* = .85) scores across both modes. Similarly, no significant differences were found across institutions (*p* = .78). Participation was voluntary, and informed consent was obtained in writing before beginning the survey. Ethics approval was obtained from the Yale School of Public Health Human Subjects Committee ref: 1112008462 with the relevant approvals sought for international research.

### Measures

#### The preliminary Pettiness Scale

The initial version consists of six items measured on a Likert-type scaling from 1 *(strongly disagree)* to 7 *(strongly agree)*. Mean scores were taken to form a score for pettiness. Higher scores indicated higher pettiness. Items are listed in Table 1.

**Table 1:** Factor loadings, communalities, means, and standard deviations for the Pettiness Scale in Study 1 (N = 1720).

Item	P	*h*^*2*^	*M*	*SD*
1) Small and unimportant things bother me.	**.66**	43.3	4.31	1.53
2) I get worked up by small things.	**.81**	65.7	3.60	1.55
3) I find fault with anything and everything.	**.76**	58.2	3.08	1.49
4) I get offended by little things people say or do.	**.82**	67.0	3.60	1.54
5) I like things done my way, even the smallest of things.	**.58**	33.1	4.00	1.50
6) Everything must be proper	.22	9.8	4.93	1.72

*Note*. Item 6 was dropped. P = Pattern coefficients; *h*^*2*^ = communalities of the measured variables; *M* = mean scores; *SD* = standard deviation. Factor loadings with values of .40 or greater are in bold.

#### The Big Five Inventory [16]

The BFI consists of 44 items measuring extraversion, agreeableness, conscientiousness, neuroticism, and openness. Participants rated themselves with respect to each statement from 1 *(strongly disagree)* to 5 *(strongly agree)*. Scores were summed with higher scores reflecting higher levels of each personality factor. Questions include “Is full of energy” (extraversion), “Is generally trusting” (agreeableness), “does a thorough job” (conscientiousness), “can be tense” (neuroticism) and “has an active imagination” (openness). All sub-scales were reliable in the current sample: extraversion (α = .73), agreeableness (α = .73), conscientiousness (α = .73), neuroticism (α = .74), and openness (α = .67).

#### The Patriotism Scale [17]

Patriotism is an appropriate construct to test the discriminant validity of the pettiness scale because pettiness and patriotism were thought to be theoretical unrelated. The Patriotism Scale measures blind (11 items) and constructive patriotism (10 items). Blind patriotism questions include “I would support my country right or wrong” and constructive patriotism questions include “All citizens should voice their opinions even if these opinions oppose the national status quo.” All items were measured on a scale from 1 *(strongly disagree)* to 7 *(strongly agree)*. Scores were summed with higher scores representing higher levels of patriotism. Blind patriotism had a reliability of α = .87 and constructive patriotism, x = .88 in the current sample.

### Results

#### Exploratory Factor Analysis (EFA)

The Kaiser-Meyer-Olkin measure of sampling adequacy index was .74, and Bartlett’s test of sphericity was highly significant, χ^2^ = 1076.26, *p* < .0001, indicating that the sample and correlation matrix were appropriate for exploratory factor analysis (EFA). Principal components analysis with varimax rotation was used. The factor structure for the variable was based on examining the scree plot, interpretability of the EFA solution, eigenvalues > 1, and a .40 cut off for factor loadings [18]. One item (‘everything must be proper’) was dropped due to low factor loading of < .40. These procedures produced a one-factor solution with five items that accounted for 53.44% of the total variance (see Table 1).

#### Internal consistency

Internal consistency was calculated using Cronbach’s alpha coefficient [19]. The alpha value of .78 for the final 5-item scale appears adequate for research purposes [20,21].

#### Construct validity

Convergent validity was established using the Big Five Inventory [16]. All correlation coefficients reached significance at *p* < .007 after correcting for multiple comparisons using the Bonferroni method [22]. As predicted, pettiness was positively correlated with neuroticism (*r* = .40, *p* < .001) and negatively correlated with agreeableness (*r* = -.36, *p* < .001), conscientiousness (*r* = -.20, *p* < .001), extraversion (*r* = -.10, *p* < .001), and openness (*r* = -.05, *p* = .038). Results suggest that pettiness is highly associated with personality dimensions. Specifically, individuals high in pettiness are likely to be high in neuroticism and low in other positive personality dimensions. Generally, the correlations were moderate and their effect sizes fell within the middle range [23]. On the other hand, as predicted, discriminant validity was evidenced through the non-significant association between pettiness and both blind (*r* = .01, *p* = .53) and constructive patriotism (*r* = -.004, *p* = .87).

## Study 1b: Further construct validation

Study 1b puts the pettiness scale through further tests of convergent and discriminant validity in a new sample. For convergent validity, we tested with stress, depression, anxiety, life satisfaction, and resilience. For discriminant validity, we tested with physical functioning.

### Participants and procedure

A new sample of 416 students from three universities in Singapore took part. Participants were 58.4% female, 70% Chinese (15% Malays; 13% Indian; 2% others), and a mean age of 20.5 (*SD* = 2.45). Procedures were similar to Study 1a except that one introductory class (from each academic discipline), instead of two, was randomly selected. The maximum potential sample is 770 and response rate is 54.03%.

### Measures

#### Depression, Anxiety, Stress Scales [24]

The DASS21 is a 21-item scale that measures a trio of negative internalizing conditions—depression, anxiety and stress (seven items per condition)—and used extensively in research across normal populations [25]. Participants rate the extent to which they experience each condition over the past week from 0 *(did not apply to me at all)* to 3 *(applied to me very much*, *or most of the time)*. Sample questions include: “I felt that life was meaningless” (depression), “I felt I was close to panic” (anxiety), “I found it hard to wind down” (stress). Scores were summed with higher scores representing greater intensity of each condition. All sub-scales are reliable in the current sample: depression (α = .83), anxiety (α = .79), stress (α = .85).

#### Life Satisfaction Scale [26]

The short 5-item scale measures global cognitive judgments of one’s satisfaction with life. Sample question include “In most ways my life is close to my ideal”, and measured on a 7-point scale from 1 *(strongly disagree)* to 7 *(strongly agree)*. Scores were summed with higher scores reflecting higher life satisfaction. The scale is reliable in the current sample (α = .84).

#### Conner and Davidson Resilience Scale (CD-RISC; [27])

Resilience was measured by the 25-item Conner and Davidson Resilience Scale. Sample questions include: “I think of myself as a strong person when dealing with life’s challenges and difficulties” and “Even when things look hopeless, I don’t give up.” Each question measured responses on a five-point scale from 0 (*not true at all*) to 4 (*true nearly all the time*). Scores were summed with higher score indicating higher resilience. This scale is reliable in the current sample (α = .91).

#### Physical functioning (SF-36; [28])

Physical functioning is measured by a 10-item scale within the SF-36 that is widely used to measure physical and mental health status. Participants are presented with 10 items that one may perform in a typical day and asked whether one’s current health status limited these activities. Sample items include “walking several blocks”, and “lifting or carrying groceries” measured on a three-point scale from 0 *(yes*, *limited a lot)* to 2 *(no*, *not limited at all)*. Higher scores indicated better physical functioning. This scale is reliable in the current sample (α = .88).

### Results

Further convergent validity was established using the DASS that measured stress, depression and anxiety. All correlation coefficients reached significance at *p* < .008 after correcting for multiple comparisons using the Bonferroni method [22]. As predicted, pettiness was positively correlated with stress (*r* = .56, *p* < .001), depression (*r* = .42, *p* < .001), and anxiety (*r* = .27, *p* < .001). Pettiness was also found to be negatively associated with life satisfaction (*r* = -.10, *p* = .009), and resilience (*r* = -.24, *p* < .001). Results indicate that individuals high in pettiness are likely to be high in internalizing conditions of stress, depression and anxiety and low in life satisfaction and resilience.

With regard to discriminant validity, as predicted, pettiness was also not associated with physical functioning (*r* = -.006, *p* = .91)—consistent with findings that personality variables were not significantly associated with physical functioning in a healthy population [29].

## Study 2: Confirmatory Factor Analysis

The purpose of Study 2 was to examine whether the Pettiness Scale has the same structure in Study 1 using confirmatory factor analysis.

### Participants and procedure

A new sample of 731 students from three universities in Singapore took part. Participants were 54.4% female, 72% Chinese (15% Malays; 10% Indian; 3% others) and a mean age of 20.9 (*SD* = 2.85). Procedures were similar to Study 1 except that one introductory class (from each academic discipline), instead of two, was randomly selected. The maximum potential sample is 1389 and response rate is 52.63%. The Cronbach’s alpha for the 5-item pettiness scale was 0.80 in this sample.

### Results

#### Confirmatory Factor Analysis

The one-factor solution distilled from exploratory factor analysis was tested using confirmatory factor analysis with AMOS 18 [30]. The model—consisting of one first order latent variable with five items as indicators—was tested against a second model with two first order latent variables representing affective (three indicators) and cognitive (two indicators) pettiness. The second model reflected the proposition of pettiness as having affective and cognitive components. Parameters were estimated by the maximum-likelihood method, which compares the fit of a hypothesized structural model to the observed variance—covariance matrix.

Multiple indices were used to evaluate model fit [31]. Traditionally, researchers used chi-square to test for model fitness. However, the exact fit hypothesis tested by chi-square is over-restrictive, sensitive to sample size and violation of normality [32]. A heuristic rule suggests that a model with a ratio of chi-square to its degree of freedom smaller than two is an acceptable fit [33]. A current trend for evaluation model fit is to use the Root Mean Square Error Approximation (RMSEA; [34]). The RMSEA measures discrepancy per degree of freedom and imposes a penalty for adding complexity to a model without substantially improving model fit. Smaller RMSEA values indicate better model fit, with values less than .05 indicating a “close fit,” between .05 and .08 corresponds to an “acceptable” fit, whereas RMSEA values larger than .10 suggest a “poor fit” [35]. The Comparative Fit Index (CFI) and the Tucker-Lewis index (TLI) measure the relative reduction in model misfit when comparing the target model relative to a baseline (independence) model. The CFI and TLI values greater than .90 have been considered an indication of an acceptable fit of the model to the observed data. This paper reported the χ^2^, CFI, TLI, and RMSEA.

Compared to the two-factor model [χ^2^ (5) = 130.60, CFI = .88, TLI = .70, RMSEA = .21], the fit indices for the one-factor model [χ^2^ (5) = 7.48, CFI = .99, TLI = .97, RMSEA = .06] were superior. Together, results of the EFA and CFA supported a one-factor model of pettiness.

## Study 3: Cross national validation

### Purpose, participants and procedure

The purpose of Study 3 is to cross validate the structure of the scale in another culture to determine cultural invariance, and examine whether the scale picks up on predicted cultural differences. We hypothesized that pettiness would be higher in Singapore than the United States because Singapore is a tight culture with high attention to detail and low tolerance for deviations, whereas the US has a loose culture with higher accommodation for deviance [12].

In the United States, we recruited 247 participants, 57% female and 85% white (mean age = 28.82; SD = 3.25) to complete an online survey measuring pettiness. The participant pool was recruited via students enrolled in a graduate class on psychometrics. The class of 13 individuals each recruited 20–25 participants by electronic means (e.g., e-mail invites). The maximum potential sample is estimated to be 325 (13 x 25) and the corresponding response rate is 76%. In Singapore, a new and independent sample of 285 participants with a mean age of 25.55 (*SD* = 2.85), 54% female and 70% Chinese were also recruited to complete a similar online survey. To keep procedures consistent, 18 students in a graduate class on Psychometrics individually sent out 20 electronic invitations, yielding a maximum potential sample of 360. The response rate is 79.17%. The Cronbach’s alpha for the US and Singapore samples are .82 and .78, respectively.

### Preliminary single-group analyses

Confirmatory Factor Analysis on the U.S. sample demonstrated an acceptable fit for the hypothesized one-factor model, χ^2^ (5, N = 251) = 13.18, CFI = .98, TLI = .94, RMSEA = .09. The Singapore sample evidenced a good fit, χ^2^ (5, N = 285) = 9.31, CFI = .99, TLI = .97, RMSEA = .05. Overall, the one-factor model of pettiness provided acceptable fit across east (Singapore) and west (U.S.A.).

### Cross cultural patterns of pettiness

As predicted, Singaporeans (*M* = 3.74, *SD* = 1.08) had significantly higher pettiness scores than Americans (*M* = 3.40; *SD* = 1.16), *F* (1, 530) = 12.23, *p* = .001. This difference appears to reflect difference in the culture rather than differences in the scale’s properties in the two countries. Equivalence of the pettiness scale in the U.S. sample was assessed and compared with a Singapore sample using sequential tests of model invariance [36]. Model 1 had no equality constraints and demonstrated a good fit, χ^2^ (6) = 18.68, CFI = .98, TLI = .95, RMSEA = .06, indicating equivalence in a one-factor model. Model 2 tested invariance of factor loadings across samples, and evidenced good fit, χ^2^ (10) = 24.17, CFI = .98, TLI = .96, RMSEA = .05. The difference between Models 1 and 2 failed to reach significance, χ^2^ (4) = 5.49, *p* = .24, providing strong support for factor loadings across cultures. Model 3 tested the invariance across factor covariances, and provided a good fit, χ^2^ (11) = 24.26, CFI = .98, TLI = .97, RMSEA = .05. The difference between Models 2 and 3 failed to reach significance, χ^2^ (1) = .09, *p* = .76, establishing invariance in factor covariances. Taken together, multiple group tests of invariance demonstrated the same one-factor structure of pettiness across both countries.

## General discussion

This is the first known study to develop and validate a scale to measure pettiness. Given the physical [5] and psychological [6] consequences, it is important to unravel the heterogeneous construct of neuroticism [3]. Pettiness reflects a facet of neuroticism that appraises trivial events with negativity.

The strong association between pettiness and neuroticism supports our theoretical proposition that pettiness is a facet of neuroticism. Of note, neuroticism is the negativity directed at both major and trivial events, while pettiness focuses specifically on the agitation induced by trivial events. Pettiness could be the most pernicious facet of neuroticism given its pervasiveness in daily life.

Initial tests suggest that the Pettiness Scale is psychometrically sound with convergent validity established through the positive associations with neuroticism, depression, anxiety and stress; negative associations with extraversion, agreeableness, conscientiousness, openness, life satisfaction and resilience. Discriminant validity was established through non-significant associations with physical functioning and patriotism.

Of broader significance, our study lays the groundwork for future research delineating how specific facets of neuroticism (e.g., pettiness) are associated with health consequences. Practically, if the pettiness-psychopathology link is established, the Pettiness Scale can be used to identify petty individuals for cognitive-behavioral intervention.

We highlight two other interesting findings. First, three studies supported a one-factor model of pettiness containing five items. Study 1 established the face, convergent and discriminant validity of the pettiness scale. Study 2 used Confirmatory Factor Analysis and produced a good fit with the one-factor model of pettiness. Study 3 established known-groups validity and found the one-factor model of pettiness to be similar in an Asian country, Singapore, and western country, United States. The cognitive-affective system theory of personality provides possible explanations for a singular factor. The theory posits that processing of cues that are significant to an individual is essentially affect-laden [37]. Consequently, cognitions resulting from the processing of those cues are themselves, highly emotional [38]. Following this rationale, considering that pettiness is defined as the tendency to get agitated by trivial events, the single factor could indicate the highly affective nature of pettiness.

Second, the cross-cultural difference in pettiness scores deserves additional attention. Findings that Singaporeans evidenced higher pettiness scores could result from their tight culture that encourages adherence to social norms. The adherence to norms permeates daily trivial events where non-conformity provokes agitation [12]. Unlike Singapore, the US is characterized as a loose culture where individuals are not as uptight.

The cross-cultural differences can also be explained by the prevention vs. promotion focus categorization. Prevention-focused cultures are concerned with the absence of *negative* outcomes, perpetuating a state of vigilance [14]. Such vigilance may explain Singaporeans’ focus on small matters and their tendency to get agitated over them. The US, on the other hand, is a promotion-focused society where the focus is on the presence of *positive* outcomes [15], hence the lower self-reported pettiness.

It should be noted, though, the differences in scores between the United States and Singapore, albeit significant, are not large. It remains unknown whether the difference in pettiness scores translates to meaningful differences in behavior and health outcomes. Perhaps, Singaporeans’ higher pettiness leads to a tendency to worry about small health behaviors such as whether they are eating enough healthy snacks. To this end, there could be health advantages to exercising some pettiness. For example, if one is petty about diet and gets agitated by the lack of fruits and vegetables, this person may make the extra effort to find and consume these foods. Future research should investigate the link between pettiness and health.

To sum up, the Pettiness Scale appears to be a promising new instrument to measure one’s tendency to get agitated over trivial matters. Given the potential health consequences of pettiness, and the brevity of the Pettiness Scale (five items), we hope that researchers consider including pettiness in future studies.

## Supporting information

S1 File Pettiness data. (RAR)
